# *In situ*-produced ^10^Be and ^26^Al indirect dating of Elarmékora Earlier Stone Age artefacts: first attempt in a savannah forest mosaic in the middle Ogooué valley, Gabon

**DOI:** 10.1098/rstb.2020.0482

**Published:** 2022-04-25

**Authors:** R. Braucher, R. Oslisly, I. Mesfin, P. P. Ntoutoume, ASTER Team

**Affiliations:** ^1^ Aix-Marseille Univ., CNRS-IRD-Collège de France-INRAE, UM 34 CEREGE, BP 80, 13545 Aix- en-Provence Cedex 4, France; ^2^ Cellule Scientifique, Agence Nationale des Parcs Nationaux, BP 20379 Libreville, Gabon; ^3^ Patrimoines Locaux Environnement et Globalisation UMR 208, IRD, MNHN, 57 rue Cuvier - C.P. 51, 75231 Paris cedex 05, France; ^4^ Muséum National d'Histoire Naturelle, UMR 7194 HNHP – MNHN, CNRS, UPVD – Alliance Sorbonne Université. Institut de Paléontologie Humaine, 1 rue René Panhard, 75013 Paris, France

**Keywords:** cosmogenic nuclides, Early Stone Age, West Central Africa, Elarmékora, Lopé national park, Gabon

## Abstract

Discovered in 1988 by R. Oslisly and B. Peyrot, Elarmékora is a high terrace that, today, is situated 175 m above the Ogooué River in the historical complex of Elarmékora, attached to the Lopé National Park in Gabon, a World Heritage site since 2007. The site yielded a small lithic assemblage, including mainly cobble artefacts embedded within the 1 m thick alluvial material. Based on geomorphological and palaeoclimatological criteria, the preliminary dating suggested an age of 400 ka. However, Elarmékora could be a key site for Atlantic Central Africa if this lithic industry can be dated absolutely. In 2018 and 2019, two field trips were organized to collect surface samples as well as samples in vertical depth profiles with the aim of measuring their *in situ*-produced cosmogenic nuclide (^10^Be and ^26^Al) content. Results suggest a surface abandonment between 730 and 620 ka ago representing a minimum age for the cobble artefacts. Concurrently, technological reappraisal of the artefacts suggests an atypical lithic industry that should, for the moment, be considered as ‘undiagnostic’ Earlier Stone Age. This age bracketing may be compared with a similar age range obtained for prehistoric occupations in Angola using the same approach. This age will place Elarmékora among the oldest evidence for the presence of hominins in western Central Africa and raises the question of a ‘West Side Story’ to early human dispersals in Africa.

This article is part of the theme issue ‘Tropical forests in the deep human past’.

## 1. Introduction

In Africa, the major contribution of Earlier Stone Age (ESA) archaeology in recent decades has been the establishment of a multidisciplinary approach combining palaeoenvironmental, palaeoanthropological and behavioural data within an increasingly reliable chronological framework. These data have allowed the reconstruction of global trends in human evolution in Africa from the first stone tool makers, 3.3 Ma ago [1], to the emergence of *Homo sapiens ca* 300 ka ago [2]. This long period, namely the ESA, is divided into two main techno-complexes based on chronological and techno-typological criteria: the Oldowan and the Acheulean. The Oldowan is a flake and core industry sometimes associated with a pebble (4–64 mm) and cobble (64–256 mm) tool component [3,4], ranging from 2.58 Ma [5] to *ca* 1.5 Ma. So far it is only reported in eastern, southern and northern Africa [6]. The subsequent Acheulean techno-complex, broadly associated with the genus *Homo*, is considered as the first technology to be widespread over the entire African continent and beyond, especially since *ca* 1 Ma [7–9]. However, once again, this techno-complex is best known from eastern, southern and northern Africa, with a large gap in our knowledge still for Central and West Africa. The Acheulean is characterized by the emergence and development of bifacial shaping, new flaking methods, large flake production (larger than 10 cm) and specific new tool types among which are large cutting tools such as handaxes and cleavers [10–14]. Some Acheulean technical patterns are believed to have persisted until the Late Pleistocene in some regions [15]. There are very few dates and geoarchaeological studies available for ESA sites in Central Africa, an area that covers the Atlantic coast to the African Great Rift Lakes, spanning from Chad to Angola [16]. It also covers a broad range environmentally, characterized by Soudano-Zambezian environments in its periphery and Guineo-Congolian environments in its centre [17].

However, a major limitation in current prehistoric research in Central Africa is its poorly resolved Pleistocene chronological and techno-cultural framework [18]. The underlying reasons for this relate both to research bias, with little specific scientific research carried out, and taphonomy, with vegetation such as tropical forest or certain climatic conditions erasing or disturbing potential evidence of past human occupation [19–21]. Also, despite the fact that several sites have suggested the presence of hominin groups in the region during the ESA [22–27], only the site of Dungo IV in Angola, located at the southern limit of Central Africa, has been dated, with an age of *ca* 600–650 ka [28]. However, this evidence is insufficient for assessing dispersal process(es) in the region, neither providing a robust palaeoenvironmental reconstruction for the specific equatorial environments of the time nor defining the hominin technical and subsistence behaviours that prevailed in the equatorial belt of Central Africa. The site of Elarmékora in the middle valley of the Ogooué River in the Lopé National Park, central Gabon, possesses numerous alluvial deposits amalgamating ESA cobble artefacts [29]. While it was discovered at the end of the 1980s, renewed consideration of the site can challenge our current understanding of early Middle Pleistocene technological variability and population dispersal within sub-Saharan Africa.

**Figure 1.**
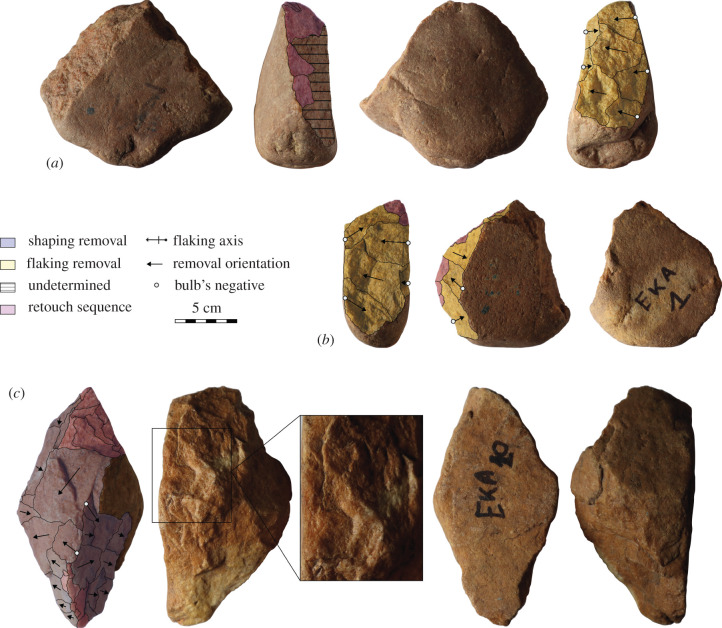
Stone artefacts from Elarmékora. A and B are core tools presenting bidirectional flaking followed by unifacial regulating retouch sequence. C is a shaped tool on angular cobble. In [30], artefact A is illustrated as no. 2, B as no. 1 and C as no. 13 (Photo credit: Isis Mesfin). (Online version in colour.)

Typologically ESA stone artefacts were found at Elarmékora in 1988 within an alluvial terrace perched 175 m above the Ogooué River (figure 1). As no source of quartz (like stone lines) can be found in the middle Ogooué valley above an altitude of 250 m, the presence of the studied stone artefacts in these deposits is puzzling.

## 2. Site presentation

The studied site is located near the Otoumbi railway station (−0.09408 S; 11.17027 E; approx. 240 m above sea level and approx. 175 m above the Ogooué River) in the northwestern part of the World Heritage site ‘Ecosystem and Relict Cultural Landscape of Lopé-Okanda’ (figure 1*a*). In this region of central Gabon, dense and well-conserved tropical rainforest coexists with relict savannah environments. A 1.2 m high exposure of the alluvial terrace can be observed near an old path formerly used in logging activities.

The Elarmékora site was probably connected to an old erosion glacis where a palaeo-Ogooué has left deposits overlying artefacts, subsequently flowing in a wider valley under arid climatic conditions that can be connected to the Middle Brunhes period [30]. Then, due to tectonic changes, the river started incising the relief, implying high denudation rates that have dismantled the old glacis and left the elevated deposits untouched. One can observe an alluvial deposit composed of rounded quartz cobbles (1–10 cm) embedded in a reddish sandy matrix and underlying a homogeneous autochthonous saprolite. Lithic artefacts have been described at the interface of the alluvial deposit and the saprolite (approx. 90 cm under the surface) [30]. These artefacts have thus been produced before their alluvial deposition at a higher elevation. Due to its dominant position and the smooth relief, one cannot observe any lateral displacements or potential arrival of colluvium from higher up that may have buried the original deposits at the site.

To better constrain the chronology of this site, possibly the oldest in Atlantic Central Africa, reinvestigations at Elarmékora aimed to identify the timing of this terrace formation, undertaken within the framework of the CAWHFI (Central Africa World Heritage Forest Initiative) programme (UNESCO). To do so, several samples were collected for dating by *in situ*-produced cosmogenic nuclides ^10^Be (T_1/2_ = 1.387 ± 0.012 My [29,31]) and ^26^Al (T_1/2_ = 0.717 ± 0.017 My [32]). This approach is now widely used but has never been attempted in such hostile conditions: at low latitude, which reduces the production rate; on a stable craton environment with potentially high inheritance implying potential difficulties for dating multiple exposure histories; and lithic artefacts close to the surface with potential continuous exposure. Usually lithic artefacts dated by burial dating are completely or mostly shielded from cosmic rays since their deposition, allowing radioactive decay of ^26^Al and ^10^Be [33–35].

Samples (quartz pebbles or coarse sand (table 1) were collected during two field campaigns in May 2018 and May 2019. In 2018, samples were collected along a vertical profile from the surface down to 140 cm (in the alluvial material from 0 to 100 cm, then in the saprolite; figure 1*b*) and three surface samples (S1, S2 and S3) were collected at the surface in the herbaceous formation. Two lithic artefacts were collected at the interface of the alluvial deposit and the saprolite to be dated (EKA 18-Outil 1 and EKA18-Outil 2). Both artefacts are quartzite cobble tools: EKA18-Outil 1 is 9 cm long and presents unifacial centripetal removals associated with a disto-lateral retouched edge, and EKA18-Outil 2 is a partially shaped tool with a pointed distal part. Regarding the technological features described in the section below, these artefacts correspond to a core tool and shaped tool, respectively. Interpretation of the 2018 results was quite difficult due to the unexpected nuclide concentration variability within the deposit (only two samples within the saprolite evidenced an exponential decrease); therefore, a second field trip was organized in 2019; the same depth profile was re-sampled but a bit deeper (195 cm). One lithic artefact, EKA19-90 has been collected at the interface of the alluvial deposit and the saprolite; this is a quartzite angular cobble. First, a distal surface is used as a flaking surface for centripetal sequence of removals. Second, a disto-lateral sequence of bifacial invasive retouch is shaping a bevel suggesting EKA 19 is a core tool.

**Table 1.** Sample positions and measured ^10^Be,^26^Al and ^27^Al concentrations. Topographic shielding factor for all samples is 1. All samples were prepared at CEREGE and measured on Accélérateur pour les Sciences de la Terre, Environnement, Risques (ASTER) accelerator mass spectrometer (AMS; see §4).

		depth	latitude	longitude	alt.	^10^Be	^26^Al	R(^26^Al/^10^Be)	natural ^27^Al
sample	type	cm	°	°	m	kat/g	kat/g		ppm
EKA18 -0	quartz pebble	0	−0.09408	11.17027	226	2312 ± 41	6663 ± 538	2.88 ± 0.24	3.59 ± 0.07
EKA18 -20	quartz pebble	20				1390 ± 29	6942 ± 354	4.99 ± 0.28	18.68 ± 0.37
EKA18 -40	quartz pebble	40				1410 ± 27	5586 ± 560	3.96 ± 0.4	16.46 ± 0.33
EKA18 -60	quartz pebble	60				776 ± 16	4777 ± 320	6.15 ± 0.43	20.1 ± 0.4
EKA18 -75-80	coarse gravel	77				912 ± 19	2187 ± 306	2.4 ± 0.34	3.3 ± 0.07
EKA18 -Outil 1	quartzite cobble	90				1576 ± 27	7183 ± 318	4.56 ± 0.22	14.33 ± 0.29
EKA18 -Outil 2	quartzite cobble	90				1077 ± 20	2786 ± 329	2.59 ± 0.31	12.29 ± 0.25
EKA18 -95	quartz cobble	95				1433 ± 29	2765 ± 223	1.93 ± 0.16	2.76 ± 0.06
EKA18 115-120	coarse gravel	117				251 ± 8	1717 ± 302	6.85 ± 1.22	22.99 ± 0.46
EKA18 -140	coarse gravel	140				147 ± 5	928 ± 179	6.33 ± 1.24	15.84 ± 0.32
EKA18 - S1	quartz cobble	0	−0.09296	11.17063	240	710 ± 14	3484 ± 456	4.9 ± 0.65	1.64 ± 0.03
EKA18 - S2	quartz cobble	0				920 ± 20	4619 ± 248	5.02 ± 0.29	3.93 ± 0.08
EKA18 - S3	quartz cobble	0				469 ± 12	2756 ± 227	5.88 ± 0.51	14.27 ± 0.29
EKA19 - 0	quartz cobble	0	−0.09408	11.17027	226	851 ± 153	4682 ± 140	5.5 ± 1.01	19.57 ± 0.39
EKA19 -20	coarse gravel	20				1253 ± 26	5583 ± 170	4.46 ± 0.16	25.02 ± 0.5
EKA19 -50	quartz pebble	50				1334 ± 27	6313 ± 213	4.73 ± 0.19	17.44 ± 0.35
EKA19 -70	quartz pebble	70				1254 ± 27	5782 ± 196	4.61 ± 0.19	15.52 ± 0.31
EKA19 -90 Q Roulé	quartz cobble	90				1349 ± 29	4508 ± 147	3.34 ± 0.13	25.52 ± 0.51
EKA19 -100	quartz pebble	100				983 ± 28	3602 ± 114	3.67 ± 0.16	15.22 ± 0.3
EKA19 -120	quartz pebble	120				295 ± 8	2014 ± 86	6.84 ± 0.34	13.14 ± 0.26
EKA19 -140	coarse gravel	140				203 ± 7	1307 ± 61	6.44 ± 0.37	16.47 ± 0.33
EKA19 -150	coarse gravel	150				195 ± 6	1315 ± 62	6.75 ± 0.38	20.91 ± 0.42
EKA19 -170	coarse gravel	170				135 ± 5	889 ± 43	6.56 ± 0.39	16.27 ± 0.33
EKA19-190-195	coarse gravel	192.5				101 ± 3	746 ± 74	7.41 ± 0.77	13.16 ± 0.26
EKA19-90-outil	quartzite cobble	90				2118 ± 39	9095 ± 272	4.29 ± 0.15	6.04 ± 0.12
EKA-HT -0	coarse gravel	0	−0.09305	11.17057	257	2169 ± 40	7478 ± 225	3.45 ± 0.12	13.94 ± 0.28
EKA-HT -30	coarse gravel	30				1039 ± 21	5336 ± 177	5.13 ± 0.2	11.36 ± 0.23
EKA-HT -50	coarse gravel	50				754 ± 23	4704 ± 185	6.24 ± 0.31	13.35 ± 0.27
EKA-HT -70	coarse gravel	70				605 ± 14	3416 ± 141	5.64 ± 0.27	11.76 ± 0.24
EKA-HT -90	coarse gravel	90				442 ± 13	2306 ± 89	5.21 ± 0.26	13.72 ± 0.27

Finally, a 1 m deep depth profile was excavated in the autochthonous formation on top of the hill, just above the alluvial deposit.

## 3. Description of stone artefacts

**Figure 2.**
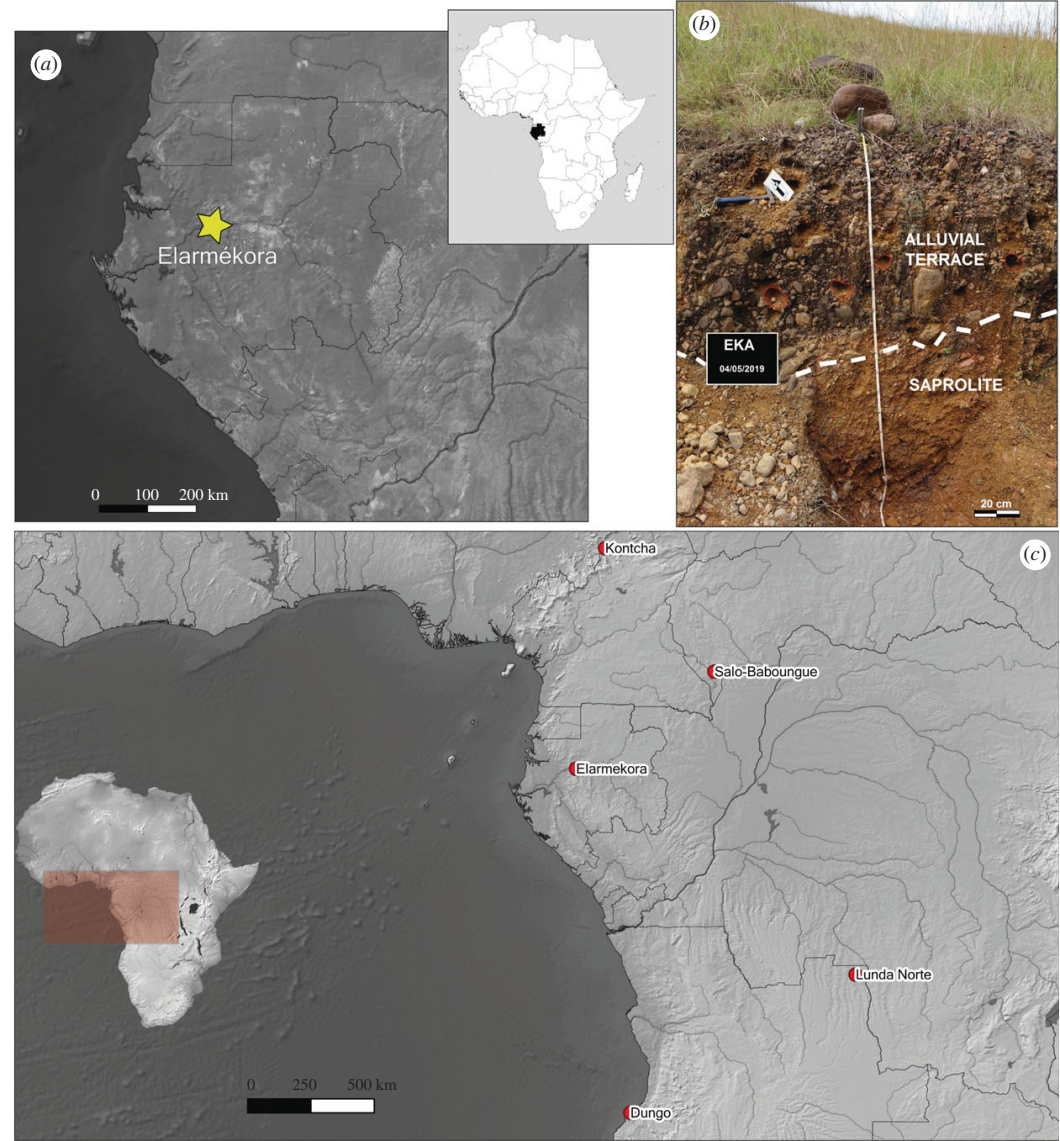
(*a*) Location of Elarmékora in centre of Gabon. (*b*) Picture of the alluvial terrace overlying the autochthonous saprolite. (Photo credit: R. Oslisly). (*c*) Map of western Central Africa and location of the sites mentioned in the text. (Online version in colour.)

**Figure 3.**
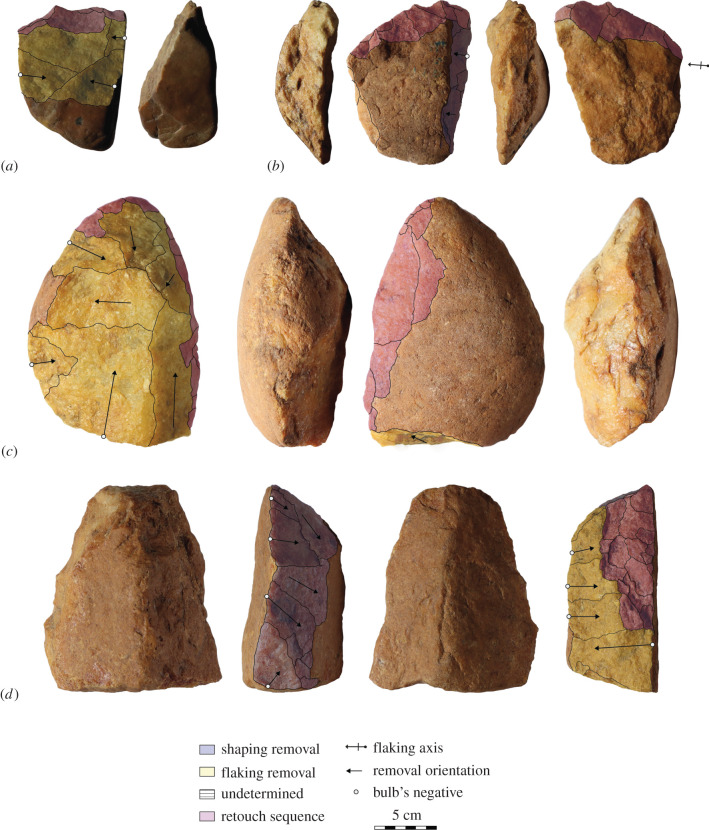
Stone artefacts from Elarmékora. (*a*), (*c*) and (*d*) are core tools. (*d*) also has a shaping sequence on the left lateral edge. (*b*) is a unifacially and partially shaped tool on a large cortical flake. In [30], artefact (*a*) is illustrated as no. 6, (*b*) as no. 7, (*c*) as no. 4 and (*d*) as no. 14. (Photo credit: Isis Mesfin.) (Online version in colour.)

The assemblage of Elarmékora is composed of 14 artefacts (figures 2 and 3) presenting clear intentional anthropic modifications: all artefacts have several regular and large removals with clear negative bulbs, and the removal orientations indicate clear flaking strategies (e.g. bidirectional, unidirectional, centripetal) [36]. These artefacts were first described as Early Acheulean in [30] based on a classic typological approach. However, it is now broadly acknowledged that ESA lithic assemblages reveal much more variable hominin behaviours than previously stated, both during the Early and the Middle Pleistocene [15,37–39], and that typological approaches provide few insights into lithic assemblage variability [40]. Consequently, we considered it necessary to revisit the artefacts and reassess their primary techno-cultural affiliation. To do so, we conducted a qualitative technological analysis and made a diacritical sketch for each artefact, grouping the removals in distinct sequences according to their orientation [41,42]. However, all of the pieces are slightly rolled, making it difficult to precisely determine the removal chronology on every piece. The dominant raw material is quartzite which was used on three types of blanks: morphologically homogeneous flat cobbles, angular cobbles and large flakes (greater than 10 cm) detached from large blocks.

Due to the small number of artefacts (*n* = 14), it is difficult to establish a robust techno-typology of the assemblage. We identified two main categories of artefacts, the shaped tools (*n* = 6)—characterized by unstandardized removals aiming to modify the shape of the blank—and the core tools (*n* = 6)—characterized by core shaping and a recurrence in the morphology and modality of removals, which may suggest intentional flake production prior to retouching [43,44]. These artefacts were identified along with one raw unmodified large and thick flake and one core presenting two sequences of unidirectional removals. All detailed measurements, weight and additional attributes are presented in a supplementary file (electronic supplementary material, table S1) along with supplementary photographs (electronic supplementary material, figure S1). Medium- to small-sized flakes and debris are absent from the assemblage. Indeed, we must consider this assemblage as influenced by the sorting of larger artefacts in the deposit. However, among the shaped tool and core tool groups, we could observe some repetitive technological and morphometrical features, suggesting an important homogeneity in the production of these artefacts. The assemblage of Elarmékora is characterized by the production of massive heavy-duty tools by using cobble blanks, taking advantage of their natural morphologies.

The shaped tools (*n* = 6, length: $\bar{X}=138.2\;\mathrm{mm}$, s.d. = 26.4; width: $\bar{X}=89.7\;\mathrm{mm}$, s.d. = 11.2; thickness $\bar{X}=59.7\;\mathrm{mm}$, s.d. = 18.3) are large tools with a trihedral or rhomboid section from the mesial to the distal and a proximal pointed tip. These tools present high indices of elongation (length/width: $\bar{X}=1.53$, s.d. = 0.17) and robustness (width/thickness: $\bar{X}=1.60$, s.d. = 0.40) demonstrating their massive character. Their overall morphology echoes the ‘pick’ tool type [45,46]. These tools are mainly shaped on angular cobbles (*n* = 4). The different flat surfaces of these blanks are used to provide several striking surfaces for shaping. Indeed, we observe that all of the shaped tools present more than two surfaces, with the exception of one cortical flake with partial unifacial shaping (figure 3*b*). It suggests that knappers were not familiar with bifacial symmetry for shaping; instead, they saw an opportunity for using the different natural flat surfaces of the angular cobbles (figure 2c). Consequently, the different surfaces of the tools are partially shaped, but we can observe the use of three or more striking surfaces. The peripheral edges are thick and rarely have retouch removals. Among the three retouched shaped tools, two have retouch scars with feather or step terminations (figure 2*c*), while the third tool has bifacial low-angle retouch. We note that thin and long cutting edges are absent from this group.

The core tools (*n* = 6, length: $\bar{X}=126.7\;\mathrm{mm}$, s.d. = 10.9; width: $\bar{X}=103.8\;\mathrm{mm}$, s.d. = 20.1; thickness $\bar{X}=61.5\;\mathrm{mm}$, s.d. = 6.5) are slightly smaller than shaped tools, but the former are larger and thicker. Also, these pieces are much broader (length/width: $\bar{X}=1.25$, s.d. = 0.23) and slightly less robust (width/thickness: $\bar{X}=1.69$, s.d. = 0.32) than shaped tools. Their shape varies from oval to quadrangular, and the section is elongated. These artefacts all show a first sequence of removals, suggesting flake production through uni- or bidirectional flaking on the lateral edge of a flat cobble. The use of two opposite large and flat cortical striking platforms may echo the use of the bipolar-on-anvil technique (figures 2*a*,*b* and 3*a,d*) [47,48]. Nevertheless, one piece (figure 3*c*) possesses a centripetal sequence of removals on a convex surface of a rounded cobble. The secondary modification of the artefact occurs through retouch sequences. Usually retouch removals aim to modify one or several peripheral cutting edges and exhibit different morphologies: abrupt, low-angle, unifacial, bifacial, invasive or short and continuous or discontinuous. This variability depicts a tendency to regularization of the initial core blank to obtain functional cutting edges.

## 4. Methods

All samples were crushed, sieved and cleaned with a mixture of HCl and H_2_SiF_6_. The extraction method [49,50] for ^10^Be and ^26^Al involves isolation and purification of quartz and elimination of atmospheric ^10^Be. Exactly 150 µl of a (3025 ± 9) ppm ^9^Be solution were added to the decontaminated quartz. Natural content of aluminum was determined by an inductively coupled plasma-optical emission spectrometer (ICP-OES) using an ICAP6500 from Thermo. Beryllium and aluminum were subsequently separated from the solution by successive anionic and cationic resin extractions (DOWEX 1X8 then 50WX8) and precipitations. The final precipitates were dried and heated at 800°C to obtain BeO and Al_2_O_3_ and finally mixed with niobium (BeO) and silver (Al_2_O_3_) powders prior to measurements, which were performed at the French accelerator mass spectrometer (AMS) National Facility, Accélérateur pour les Sciences de la Terre, Environnement, Risques (ASTER), located at CEREGE in Aix-en-Provence. Beryllium data were calibrated directly against the STD11 standard [51] with a ^10^Be/^9^Be ratio of (1.191 ± 0.013) × 10^−11^. Aluminum measurements were performed against an in-house standard called SM-Al-11 with ^26^Al/^27^Al = (7.401 ± 0.064) × 10^−12^, which has been cross-calibrated against the primary standards certified by a round-robin exercise [50]. Analytical uncertainties (reported as 1*σ*) include uncertainties associated with AMS counting statistics, AMS external error (0.5% for ^10^Be), chemical blank measurement and, regarding ^26^Al, ^27^Al measurements.

Measurements of chemically processed blank yield ratios on the order of (2.0 ± 0.75) × 10^−15^ for ^10^Be and (2.0 ± 2.0) × 10^−15^ for ^26^Al. A sea-level high-latitude spallation production rate of 4.02 ± 0.32 at. g^–1^ a^–1^ [52] was used and scaled using [53] polynomials. The ^26^Al/^10^Be production ratio induced by the standardization used at ASTER is 6.61 ± 0.50.

The general equation used to model ^10^Be and ^26^Al concentrations considering the three types of particles involved is given by equation (4.1):

4.1 $$\begin{array}{rl} N(x,\;\varepsilon ,\;t)= & \;\frac{P_{n}.\;e^{-\rho x/\Lambda_{n}}.\;(1-e^{\left(-t((\rho \varepsilon /\Lambda_{n})+\lambda )\right)})}{\frac{\rho \varepsilon }{\Lambda_{n}}+\lambda }\\ & +\frac{P_{\mathrm{slow}}.\;e^{-\rho x/\Lambda_{\mathrm{slow}}}.\;(1-e^{-t((\rho \varepsilon \Lambda_{\mathrm{slow}})+\lambda )})}{(\rho \varepsilon /\Lambda_{\mathrm{slow}})+\lambda }\\ & +\frac{P_{\mathrm{fast}}.\;e^{-\rho x\Lambda_{\mathrm{fast}}}.\;(1-e^{-t((\rho \varepsilon /\Lambda_{\mathrm{fast}})+\lambda )})}{(\rho \varepsilon /\Lambda_{\mathrm{fast}})+\lambda },\\ & +N(0,\varepsilon_{2},\infty )\;.\;e^{-\lambda t} \end{array}$$

where *P_n_*, *P*_slow_ and *P*_fast_ are the production of neutrons, stopping and fast muons respectively, *ρ* is the material density, *ε* is the denudation rate, *t* is time, *Λ_n_*, *Λ*_stop_ and *Λ*_fast_ are the attenuation lengths of neutrons (150 g cm^−2^) and stopping (1500 g cm^−2^) and fast muons (4320 g cm^−2^), respectively. The term $N(0,\;\varepsilon_{2},\;\infty )$ is a potential inheritance coming from a previous exposure at steady state (T = infinite) and with a denudation *ε_2_*. This denudation *ε*_2_ will be referred to in the following as a palaeo denudation rate; since the samples might have undergone different exposure histories before the deposition event, the term *ε*_2_ is allowed to vary among samples. *λ* is the radioactive decay constant (*λ* = ln2/half-life). Muon contribution scheme follows [54].

## 5. Results and discussion

All data are presented in table 1. Regarding the depth profile samples EKA18 and EKA19, one can observe two groups of data delimited by the interface between the alluvial deposit and the saprolite (figure 1*b*). Within the saprolite (2018 samples EKA18-115-120 and EKA18-140 extended with 2019 samples EKA19-120, EKA19-140, EKA19-150, EKA19-170 and EKA19-190-195), the concentrations clearly follow the expected exponential decrease due to the attenuation of cosmic ray particles in the Earth's matter. In the first metre, these attenuation lengths are 156(+13/−12) g cm^−2^ for ^26^Al and 145(+8/−6) g cm^−2^ for ^10^Be in quartz for neutrons [55]. For EKA19 samples within the saprolite, using a mean density of 2.4 g cm^−3^ deduced from individual density measurements, the experimental apparent attenuations are approximately 162 g cm^−2^ for ^10^Be and approximately 169 g cm^−2^ for ^26^Al. This thus unambiguously implies that the studied saprolite was always exposed within the first metres and therefore was never deeply buried by the alluvial deposits.

**Figure 4.**
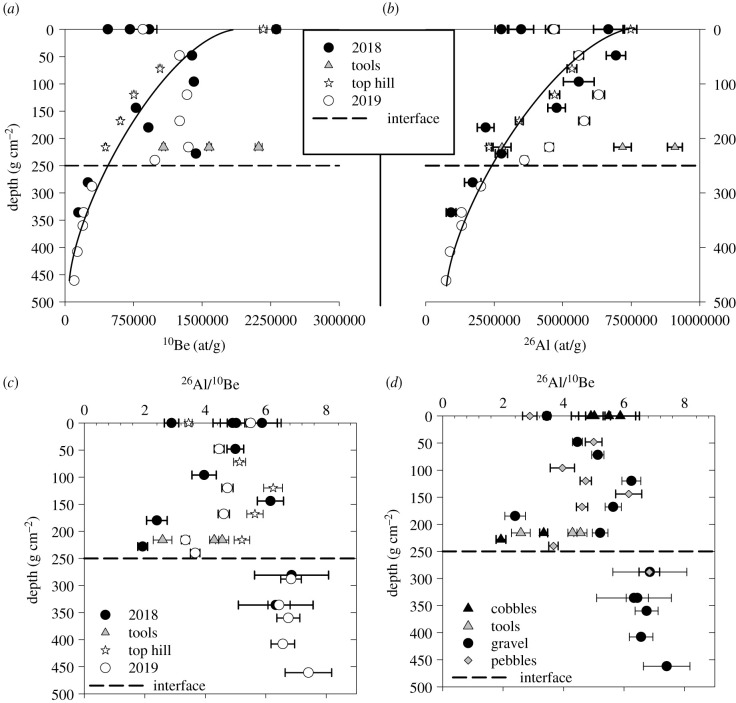
^10^Be (*a*), ^26^Al (*b*) and ^26^Al/^10^Be ratio (*c*,*d*) as a function of depth for EKA18, EKA19 and TH samples. (*d*) Presents the ratios as a function of sample types (Cobbles (including tools), pebbles and gravels). Dashed line represents the interface between the alluvial deposit and the saprolite (figure 2*b*), and the black line shows the exponential decrease due to neutron attenuation in the penetrated material (see §5).

In the alluvial deposit above the interface, concentrations are, at first glance, more randomly distributed for samples from both the 2018 and 2019 field campaigns. This was one reason behind sampling the top hill depth profile at a slightly higher elevation than the alluvial terrace, but in an area without any signs of the deposit that may be the cause of the variability. In fact, at this position, the expected exponential decrease is observed (stars in figure 4 in the two upper panels). Moreover, when considering the concentrations of the EKA-TH profile, one can see that the exponential decrease of EKA-TH sample concentrations can be extended to the deeper ones within the saprolite (samples mentioned above); this is represented by the black lines in figure 4 in the two upper panels.

Considering ^26^Al/^10^Be ratios, one can observe (figure 4*c* and *d*) that they are quite homogeneous within the saprolite and more scattered above the interface, with some values that may indicate a complex burial history (EKA18-0; EKA18-Outil2, EKA18-95).

This confirms again that alluvial disturbance has affected only the upper first metre of the studied surface. Finally, one can also observe in figure 4 that all sample concentrations above the interface are: (i) higher than the interface concentration (approx. 440 kat g^−1^ and 2300 kat g^−1^ for ^10^Be and ^26^Al respectively) and (ii) lower than the top surface concentration (approx. 2300 kat g^−1^ and 6800 kat g^−1^ for ^10^Be and ^26^Al, respectively), with the exception of EKA19-90-outil ^26^Al concentration. These observations suggest that all samples may have thus evolved *in situ* and that the first metre has been subsequently perturbed that may be potentially link to biological activity [56,57] or, may be the results of a strong event that has dismantled an old indurated ferricrust whose relicts can be observed in the field (see electronic supplementary material, figures S2 and S3).

All these observations being made, the big challenge is to date this surface in order to have at least a minimum age for the found artefacts.

Based on our data descriptions, it was decided that four models should be performed to better bracket the most probable exposure age. All models are based on the depth profile approach [58,59]. Although the approach of Hidy *et al*. [59] has been developed on amalgamated samples, it can also be applied on single clasts even though inheritance may be less homogeneous for clasts. Using this single nuclide approach for the first time is interesting to see if both ^10^Be and ^26^Al outputs agree.

The Monte Carlo approach of [59] has thus been performed on samples that lie on the exponential decrease shown on figure 4 considering: (i) the depth profile from saprolite samples only, (ii) a depth profile considering the maximum of samples that are near the exponential decrease curve, (iii) the ‘top hill’ depth profile samples and finally (iv) a composite profile grouping the saprolite and the ‘top hill’ samples (a and c).

Outputs can be observed in table 2; all exposure ages (minimum or maximum) determined by ^26^Al are always lower than those determined by ^10^Be. Considering ^10^Be and ^26^Al separately, the overall maximum and minimum ages for the EKA profiles (alluvial deposit and/or saprolite samples, ‘top hill’ profile not included) range from 456.4 to 1017 ka.

**Table 2.** Model outputs. The first number is the age (ka) and the second the denudation rate (m Ma^–1^). For all simulations and based on the considered samples, inheritance is negligible.

profile	^10^Be		^26^Al		^10^Be and ^26^Al	
	min (T/*ε*)	max (T/*ε*)	min (T/*ε*)	max (T/*ε*)	min (T/*ε*)	max (T/*ε*)
saprolite sample	663/0	999/0.31	470/0	526/0.05	627/0	720/0.2
max. samples	674/0	1017/0.44	460/0	558/0.23	620/0	730/0.25
hill top	772/0	1179/0.4	457/0	988/0.98	512/0.9	infinite/0.95
composite (saprolite samples + hill top)	772/0	1180/0.4	482/0	529/0.1	700/0.22	1018/ 0.72

For the same selected profiles, a model based on equation (4.1), combining the two nuclides has been also performed using an Excel spreadsheet. For all samples, a unique exposure time (*t*) and a unique denudation rate (*ε*) after the deposition event have been considered, but palaeo denudation rates (*ε*2) were considered as free parameters for each sample. Uncertainties were determined following [60] using the χ^2^ plus one.

Combining the two nuclides allows a reduction in the time span from 620 ka to 730 ka and denudation rates from 0 to 0.25 m Ma^−1^ for the alluvial deposit and/or saprolite samples.

For all simulations, inheritance can be neglected when considering samples close to the exponential decrease.

Considering the three lithic artefacts totally shielded from cosmic rays, their concentrations yield minimum burial ages (no post production) close to 300 ka for EKA18-Outil 1 and EKA19-90-Outil and close to 1.4 Ma for EKA18-Outil2 with palaeo-denudation rates within the range of 0.45–0.7 m Ma^−1^. EKA18-Outil 2 clearly has a complex exposure history or was produced on a previously buried cobble.

One has to be resigned and accept the fact that the minimum age of these artefacts is that of the deposit they belong to, i.e. 620 ka, and that no direct age can be determined.

The same dating difficulties arose in Angola [28], where lithic remains were found buried in a sandy matrix whose age was determined to be close to 650 ka, contemporaneous with the Elarmékora site. However, the Angolan artefacts were buried deeper (approx. 3 m) and have buried ages ranging from 0.7 to 2 Ma but as for Elarmékora, the minimum age to be trusted is the matrix age they belong to.

While few archaeological studies have been done in western Africa, the minimum age of 620 ka falls just after the mid-Pleistocene transition [61,62], coincident with the onset and intensification of high-latitude glacial cycles [63]. These climatic changes, probably coupled with tectonic activity, have been identified in other parts of Africa and seem to have impacted faunal populations [64–68].

When considering the technological patterns of the Elarmékora lithic assemblage, we face a difficulty in its classification. On the one hand, the large flake production evidenced by two artefacts and the presence of a pick tool-type may echo the Acheulean techno-complex, which is contemporary to Elarmékora and more broadly prevails in sub-Saharan Africa during the early Middle Pleistocene [10–12,46,69]. On the other hand, some typical Acheulean technical patterns such as large cutting tools, bifacial shaping and specific tool types such as cleavers, handaxes or polyhedra are absent from the Elarmékora assemblage. A shaping strategy is present, but it never involves the use of bifacial symmetry for guiding the reduction sequence. In addition, the types of flaking strategies identified at Elarmékora may not be associated with a specific time period or any techno-cultural entity as these are pan-chronological features. Overall, in the Elarmékora assemblage we identified both general technological affinities with the Acheulean techno-complex and specific local technical features, such as exploiting the natural volumetric advantages of the pebbles, the ‘multifacial’ shaping and the close relationship between cores and pebble tools. Consequently, due to these specific patterns and to the small size of the assemblage, we now may consider the lithic technology of Elarmékora as an ‘undiagnostic ESA’. Finally, this site provides data on ESA technology in the equatorial belt of Central Africa which may, in the future, contribute to refining our understanding of the specific role of equatorial regions in human evolution [70,71].

**Figure 5.**
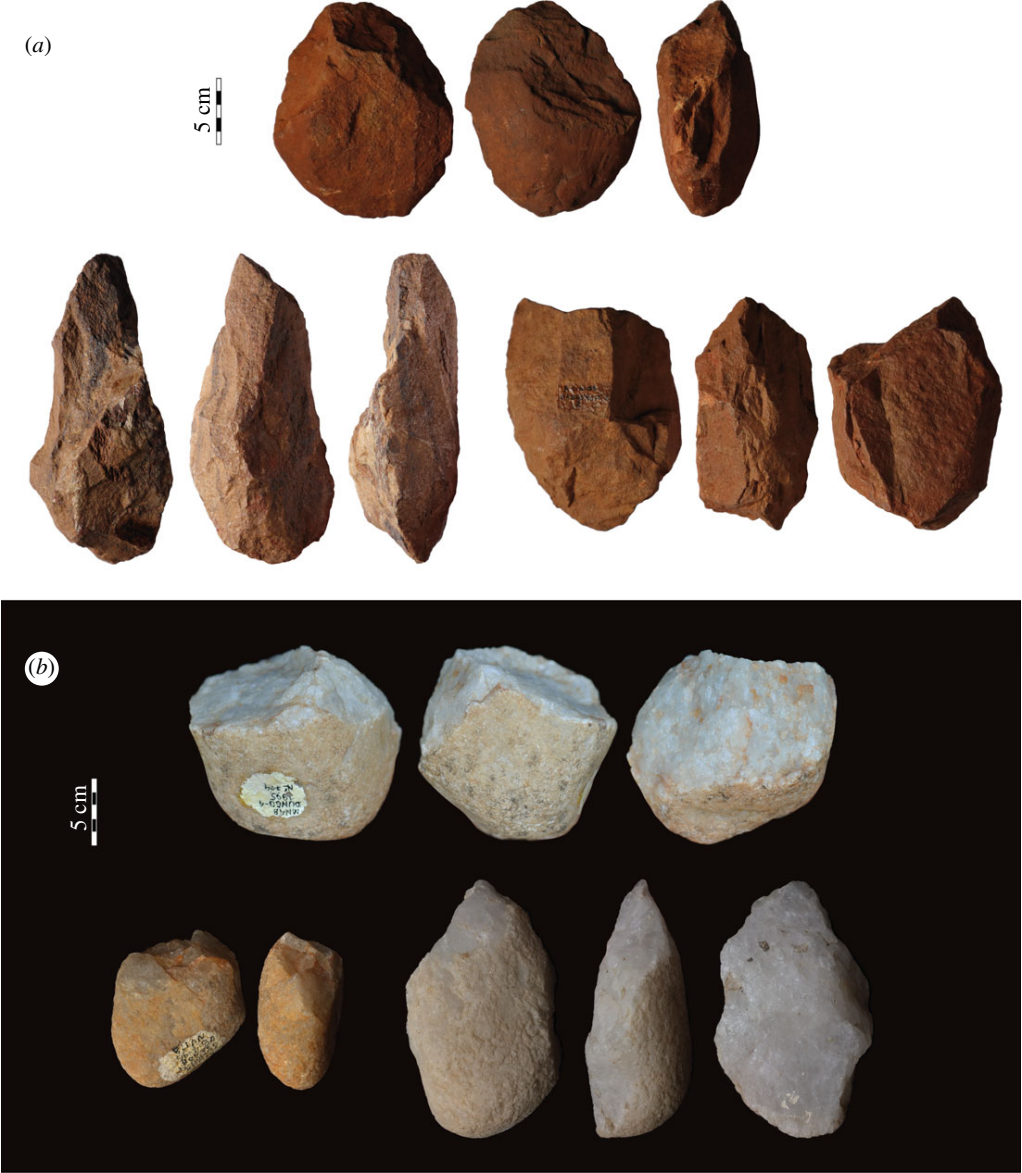
(*a*) ESA artefacts from Baboungué, Central African Republic. (*b*) ESA artefacts on pebbles and cobbles from Dungo IV. (Photo credit: Isis Mesfin.) (Online version in colour.)

So far, only the site of Dungo in Angola presents ages that converge with those of Elarmékora, dated by cosmogenic nuclides to *ca* 600–650 ka. The technological patterns of Dungo also suggest a dominance of pebble and cobbles tools (figure 5*b*) along with some shaped tool production [72,73]. Similar patterns have been reported from a number of undated ESA sites in western Central Africa (figure 1*c*), among which are the Lunda-Norte sites in northeastern Angola [74]. Comparable technological trends have been observed on other Central African ESA sites such as Baboungué in the Sangha River Basin in Central African Republic (figure 5*a*) [23] and Kontcha in Cameroon [75]. While these remain undated, the site of Kontcha offers good characteristics for applying the same cosmogenic dating methods as those used here, since it is located on a high alluvial terrace covered with a lateritic cuirass that is elevated more than 35 m above the Mayo Deo River.

Despite the current lack of hominin fossils in western sub-Saharan Africa, the convergence of the Elarmékora ages with the sites of Dungo in Angola is remarkable because for the first time we can glimpse a new hominin dispersal scenario. To confirm this ‘West Side Story’, more dateable sites are necessary to refine the chronology of early human dispersals and to provide inter-site lithic comparison to better understand local technical trajectories during the Middle Pleistocene.

## 6. Conclusion

The significance of this discovery lies in the fact that it is the first time that an ESA site has been dated on the Atlantic edge of the Congo Basin, a vast region where research is not developed due to dense forest cover that does not promote accessibility and complicates logistics.

Despite hostile climatic conditions that prevent the good conservation of open-air Pleistocene sites, the lithic artefacts discovered in the alluvial deposit of Elarmékora have been dated as old as 650 ka at a minimum by the use of cosmogenic ^10^Be and ^26^Al pairs. This minimum age falls just at the end of a major climatic change, the mid-Pleistocene transition, observed throughout the world. The atypical lithic assemblage of Elarmékora points toward a specific ESA technology in western Congo Basin. Even though the assemblage needs to be enlarged, we presented technical specificities that raise questions on the origins of these populations, on the relationships between the contemporary Acheulean technology that prevails in a large part of Africa during the mid-Pleistocene transition and on the potential adaptation of the tool-kits in the equatorial belt.

This study confirms the antiquity of the hominin presence in western Central Africa more than 3500 km away from the closest hominin fossil sites in South Africa. It shows a tremendous advance in our knowledge of the evolution of our ancestors that could upset the established models and could provide the first evidence of a ‘West Side Story’ for early hominin dispersal within Africa.

## Data Availability

Data are presented in table 1, and AMS standardization is explained in §4. All artefacts collected at Elarmékora are stored at IPH-MNHN laboratory in Paris (contact person: R. Oslisly (oslisly.richard@orange.fr)).
